# Simultaneous Multiplexed Quantification of Banned Sudan Dyes Using Surface Enhanced Raman Scattering and Chemometrics

**DOI:** 10.3390/s22207832

**Published:** 2022-10-15

**Authors:** Taghrid S. Alomar, Najla AlMasoud, Yun Xu, Cassio Lima, Baris Akbali, Simon Maher, Royston Goodacre

**Affiliations:** 1 Department of Chemistry, College of Science, Princess Nourah bint Abdulrahman University, Riyadh 11671, Saudi Arabia; 2 Centre for Metabolomics Research, Department of Biochemistry and Systems Biology, Institute of Systems, Molecular and Integrative Biology, University of Liverpool, Biosciences Building, Crown Street, Liverpool L69 7ZB, UK; 3 Department of Electrical Engineering and Electronics, University of Liverpool, Brownlow Hill, Liverpool L69 3GJ, UK; 4 Department of Engineering and System Science, National Tsing Hua University, Hsinchu 30013, Taiwan

**Keywords:** Raman, SERS, Sudan dyes, chemometrics, PLS-R, sustainable chemistry

## Abstract

Azo compounds such as the Sudan dyes I–IV are frequently used illegally as colorants and added to a wide range of foods. These compounds have been linked to a number of food safety hazards. Several methods have been proposed to detect food contamination by azo compounds and most of these are laboratory based; however, the development of reliable and portable methods for the detection and quantification of food contaminated by these chemicals in low concentration is still needed due to their potentially carcinogenic properties. In this study, we investigated the ability of surface enhanced Raman scattering (SERS) combined with chemometrics to quantify Sudan I–IV dyes. SERS spectra were acquired using a portable Raman device and gold nanoparticles were employed as the SERS substrate. As these dyes are hydrophobic, they were first dissolved in water: acetonitrile (1:10, *v/v*) as single Sudan dyes (I–IV) at varying concentrations. SERS was performed at 785 nm and the spectra were analyzed by using partial least squares regression (PLS-R) with double cross-validations. The coefficient of determination (*Q*^2^) were 0.9286, 0.9206, 0.8676 and 0.9705 for Sudan I to IV, respectively; the corresponding limits of detection (LOD) for these dyes were estimated to be 6.27 × 10^−6^, 5.35 × 10^−5^, 9.40 × 10^−6^ and 1.84 × 10^−6^ M. Next, quadruplex mixtures were made containing all four Sudan dyes. As the number of possible combinations needed to cover the full concentration range at 5% intervals would have meant collecting SERS spectra from 194,481 samples (21^4^ combinations) we used a sustainable solution based on Latin hypercubic sampling and reduced the number of mixtures to be analyzed to just 90. After collecting SERS spectra from these mixture PLS-R models with bootstrapping validations were employed. The results were slightly worse in which the *Q*^2^ for Sudan I to IV were 0.8593, 0.7255, 0.5207 and 0.5940 when PLS1 models (i.e., one model for one dye) was employed and they changed to 0.8329, 0.7288, 0.5032 and 0.5459 when PLS2 models were employed (i.e., four dyes were modelled simultaneously). These results showed the potential of SERS to be used as a high-throughput, low-cost, and reliable methods for detecting and quantifying multiple Sudan dyes in low concentration from illegally adulterated samples.

## 1. Introduction

Sudan dyes I–IV are a class of azo dyes, which fall into a family of fat-soluble compounds and are typically used for various industrial and scientific applications. Due to their bright vibrant colours and stability, these dyes can be used in plastic, fuel, gasoline, diesel, oil, textile, solvents, polymer dyeing, cosmetics [1,2,3] and particularly within the food industry [4]. They have been used for food colouring and adulteration of (e.g.,) chili [5] and palm oil [6] due to their low cost and widespread availability. Sudan dye levels are typically reported to be in the low mg/kg range [7]. These molecules are poorly soluble in water and possess π-π systems and electron-rich atoms [8]. According to Gurr, the solubilities of Sudan I–IV are quite close to each other [9]. The diazo bonds in Sudan dyes can be broken down to generate aromatic amines, which increases the risk of cancer in humans [10,11], allergic skin reactions and genetic defects [12]. Therefore, these substances are prohibited in food industry.

There has been an increasing number of publications on the analyses of trace levels of various chemical hazards in different foods, such as restricted or banned food additives [13] and veterinary drugs [14], or fruits and vegetables contaminated with pesticides [15]. Many analytical methods have been developed to detect Sudan dyes in foods. Ultra-high performance supercritical fluid chromatography (UHPSFC) was used accurately to separate and quantitatively determine Sudan dyes in chili products [16]. High-performance liquid chromatography (HPLC) is the most often used technique for detecting Sudan dyes, as it has the ability to detect multiple Sudan dyes at the same time with great sensitivity and repeatability using different detection systems including UV–vis, electrochemical, and increasingly mass-spectrometry [7]. Although these methods have been successful to detect Sudan dyes in complex mixture, HPLC requires complex and time-consuming sample preparation procedures in order to extract and purify Sudan dyes from food, followed by lengthy chromatography [17]. Electrochemical methods [18] and fluorescence-based techniques can also be used for detecting Sudan dyes, however, these methods are not effective at detecting all four Sudan dyes [19].

Previous studies have shown that Sudan dyes at low concentration can be identified by surface-enhanced Raman scattering (SERS) [20]. However, most studies using SERS to analyze Sudan dyes have only focused on Sudan I, or have evaluated the enhancement effects of a substrate on standard Sudan dye solutions [21,22]. The majority of more recent work has concentrated on theoretical and technological development [23,24]; however, a few studies have been conducted adopting qualitative methods [25,26,27], whereas others have involved multivariate quantitative analysis [22]. To date no one has reported the simultaneous multiplexed quantification of all four Sudan dyes.

This work aims to develop a gold nanoparticle-based SERS approach, employing a portable Raman spectrometer, to detect and quantify Sudan I–IV. We shall show that it is possible to simultaneous quantify rapidly all four Sudan dyes within mixtures containing multiple Sudan dyes at different concentrations.

## 2. Materials and Methods

### 2.1. Chemicals and Solvents

In this study, Sudan I, II, III and IV, chloroauric acid, trisodium citrate (purity 99.9%), sodium chloride and acetonitrile were all analytical grade, and purchased from Sigma Aldrich Ltd. (Gillingham, UK).

### 2.2. Preparation of Citrate-Reduced Gold Colloid

Citrate-reduced gold colloid (CRGC) was prepared based on the Lee and Meisel method [28]. Synthesis was performed in a fume hood and all glassware were soaked in aqua regia (HNO_3_:HCl 1:3 *v/v*) overnight prior to use. Chloroauric acid (0.240 g, HAuCl_4_) was dissolved into 500 mL of deionized water then brought to a boil after setting the temperature of stirrer plate to 150 °C. To ensure continuous mixing a magnetic stirrer was added. Next, 50 mL of trisodium citrate solution (containing 1.0 g Na_3_C_6_H_5_O_7_ dissolved in 100 mL deionized water) was added to the chloroauric acid/water solution. The solution was then left for 1 h while constantly being stirred and heated. To achieve successful nanoparticle formation, the colour of solution should change from colourless to a dark red/purple, and this was observed. The final CRGC were characterized using UV-Vis spectrophotometer on a Jenway 7200 Visible 72 Series Diode Array Scanning Spectrophotometer (Cole-Parmer Ltd., Staffordshire, UK). Spectra from 335–800 nm are shown in Figure S1. Scanning electron microsopy (SEM) was used to acquire micrographs using a Hitachi S-4800 microscope operating at a voltage of 1 kV. Micrographs obtained from CRGC at different magnifications are illustrated in Figure S5.

### 2.3. Preparation of Sudan Dye Solutions

A series of standard solutions with seven different concentrations ranging from 0.03 mM to 0.002 mM for each of four Sudan dyes were prepared from diluting stock solutions prepared by dissolving these azo dyes in 1:10 (*v/v*) water: acetonitrile. In addition, a total number of 90 mixtures (Table S1) with different concentrations of the four Sudan dyes were prepared using Latin Hypercube sampling (LHS) [29] experimental design with the aim of ensuring good coverages of different combinations of concentrations of all four analytes in the mixtures.

### 2.4. SERS Measurements

For SERS 250 μL gold colloid was first mixed with 250 μL each sample, followed by the addition of 50 μL NaCl (0.5 M) as the aggregating agent. A DeltaNu Advantage benchtop Raman spectrometer (Delta Nu, Laramie, WY, USA), equipped with a laser wavelength of 785 nm delivering a power density on the sample of ∼60 mW was used to collect SERS spectra, Raman device was calibrated daily using toluene. The spectral range was between 400–2000 cm^−1^, the exposure time was 30 s and three technical replicate spectra were collected from each sample.

### 2.5. Data Analysis

Spectra were first baseline corrected using asymmetric least squares, subsequently smoothed via Savitzky−Golay and then normalized using vector normalization. These spectral data were first subjected to principal component analysis (PCA) [30]. PCA is an unsupervised method which enables visualization of high dimensional data in low dimensional space [31].

Partial least squares regression (PLS-R) [32] was then employed for quantitative analysis of Sudan dyes. For PLS-R modelling, only standard normal variate (SNV) [33] pre-processing was applied. For single dye solutions (simplex mixtures), the models were validated using *k*-fold double cross-validation [34] for single dye solutions where *k* equals the number of different concentrations (i.e., 7). The limit of detection was estimated from the results of PLS-R model using the method as described by Blanco et al. [35]. From the 90 mixtures containing all four Sudan dyes PLS-R was also used for modelling and this was performed in two different ways:

(i) Four separate PLS-R models (based on PLS1) were generated to predict each of the four dyes in the quadruplex individually. A single output Y-variable encoded the concentration of the dye in each of the PLS models.
(ii) A single PLS-R model was generated to predict all four dyes simultaneously where 4 outputs (Y-data) were used (in PLS2) to encode the concentration of each of the 4 dyes in the quadruplex mixtures.

Bootstrap resamplings [36,37] were used to validate the PLS-R models from these quadruplex mixtures. For (i) each of the 4 PLS-R models used 1000 bootstraps, while for (ii) where PLS-R aimed to predict all four dyes simultaneously 10,000 bootstraps were calculated.

All data processing was carried out in Matlab R2021b (MathWorks Inc., Natwick, MA, USA) and the PCA and PLS code are freely available via our GitHub respository (https://github.com/Biospec/).

## 3. Results and Discussion

**Figure 1:**
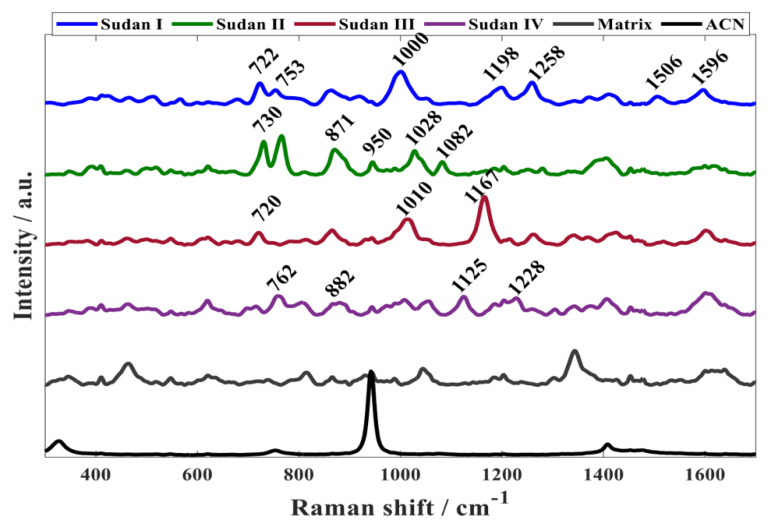
Representative SERS spectra of Sudan I (0.048 mM), II (0.036 mM), III (0.028 mM), and V (0.026 mM), Matrix (this is the aggregated gold nanoparticles with no dyes present so shows the citrate background) and the acetonitrile solvent (ACN).

**Figure 2:**
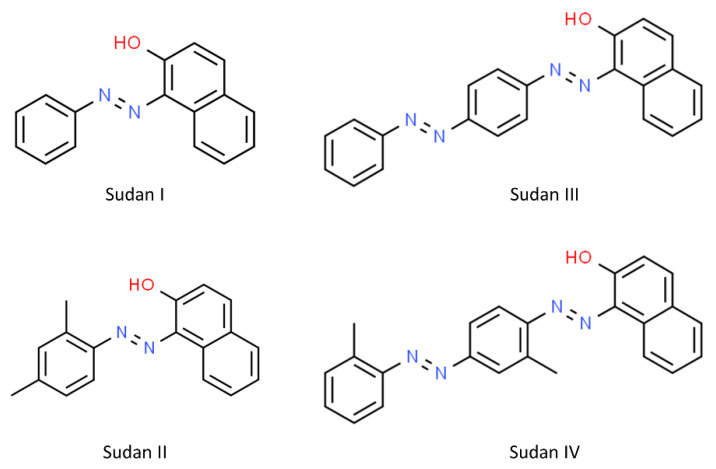
Molecular structures of Sudan dyes I–IV. These structures were taken from ChemSpider (https://www.chemspider.com).

Figure 1 shows the fingerprint region (300–1700 cm^−^^1^) of SERS spectra obtained from Sudan dyes I–IV. Although Sudan II, III and IV are derived from 1-phenyl-azo-2-naphthol (Sudan I) (Figure 2), the SERS spectra acquired from the different Sudan dyes are clearly different, which is probably due to the association of different parts of these molecules with the gold nanoparticles, which is likely to be through the diazene functional group as the N=N can become positively charged and associate with the negative charge from the citrate that caps the gold nanoparticles.

### 3.1. Quantification of Individual Sudan Dyes

As the dyes are hydrophobic stock solutions of each of the Sudan dyes at 0.048 mM, these were made and diluted in a mixture of water and ACN (1:10 *v/v*).

**Figure 3:**
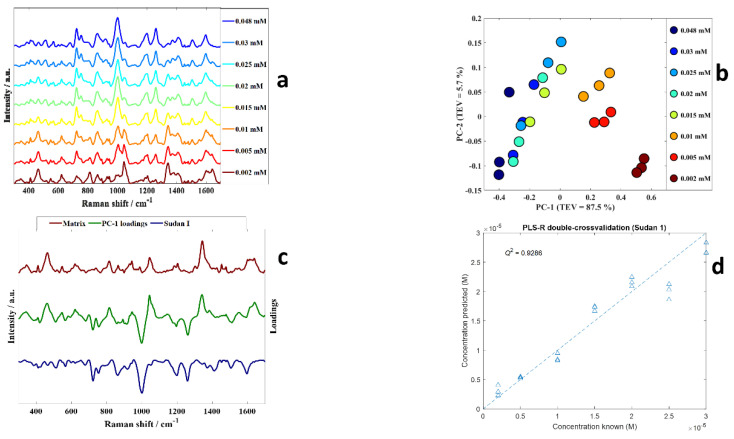
(**a**) SERS spectra of Sudan I shows different concentrations and spectra are offset for clarity; (**b**) PCA scores plot from SERS spectra from Sudan I, the colours represent the concentrations, and the details are provided within the figure; (**c**) PC1 loadings plot (green), matrix (aggregation agent (0.5 M NaCl) and gold nanoparticles) (brown), and Sudan I (blue, multiplied by −1 (i.e., inverted) for clarity); (**d**) PLS−R predictions of Sudan I, these models used double-cross validation.

Taking Sudan I as an example, samples with varying concentrations of Sudan I (0.03 mM, 0.025 mM, 0.02 mM, 0.015 mM, 0.01 mM, 0.005 mM and 0.002 mM) were made and the SERS spectra acquired (Figure 3a). The intensities of peaks in SERS spectra of Sudan I dyes decreased with the concentration as shown in Figure 3a. For example, the intensities of the peaks at ~722, 753, 1000, 1198, 1258 and 1506 cm^−1^ which can be ascribed to Sudan I were lower in the 0.002 mM solution compared to 0.048 mM Sudan I. Changes in SERS intensities were caused by the number of molecules close to the local plasmonic field, the interactions between the analyte molecules and the gold nanoparticles surface, as well as potentially being affected by adsorption sites and orientations of the dyes on the surface [38,39].

PCA scores plot of the dilution series from Sudan I (Figure 3b) shows a clear trend along PC 1 axis from positive to negative scores with increasing concentrations, the linear range which for Sudan I is 0.048–0.002 mM. This means the dominant effect is the dilution of the analyte as PC1 is extracted to explain the most natural variance in these data. Inspection of the PC1 loadings plot (Figure 3c) showed negative loadings associated with peaks related to Sudan I and positive loadings to signatures associated with the matrix (the ACN solvent and citrate on the surface of the gold nanoparticles (AuNPs)). For clarity in this plot the matrix of the AuNPs alone without any analyte is plotted in brown above the positive PC1 loadings spectrum, showing peaks that are from the matrix being dominant in positive PC1 loadings. While on the negative side of the PC1 loadings, we have plotted the inverted spectrum of Sudan I. There is clear congruence between peaks that are from Sudan I and the negative PC1 loadings plot.

PLS-R was used to establish quantitative models for predicting the level of Sudan I in these diluted samples. In this process the input data were the SERS spectra and the output the concentration (M) of Sudan I. As described in the Section 2 *k*-fold double cross-validation was used to assess the reproducibility and validity of these models. The results of the PLS-R predictions of the concentration of Sudan in terms of the predicted versus known concentrations of Sudan I dyes are presented in Figure 3d where good predictive accuracies can be seen for this Sudan I standard dilution series. We calculated figures of merit where *Q*^2^ is the goodness-of-fit of the test set (cross validation) predictions and the closer to 1 the better the fit. We also calculated the error in the predictions from the know levels using root mean squared error on the cross validation (RMSECV) samples, as well as the limit of detection (LoD) as also described in the Section 2. For the PLS-R model for Sudan I the *Q*^2^ was 0.9286, RMSECV was 2.5616 × 10^−6^ M and the estimated LoD was 6.27 × 10^−6^ M. This LoD of Sudan I corresponds with the LoD in a study conducted by Dao and colleagues [20].

Having established that SERS with PLS-R was able to quantify Sudan I in a dilution series, PCA and PLS-R were also applied to SERS spectral data acquired from varying concentrations of Sudan II, III and IV (Figures S2–S4, respectively) where the linear regions were established as–Sudan II: 0.036–0.004 mM; Sudan III: 0.028–0.002 mM; Sudan IV: 0.026–0.002 mM. Trends associated with SERS spectra acquired from solutions with varying concentrations were also observed in the PCA scores plot of Sudan II–IV dyes along PC 1 axis (Figures S2–S4). In addition, the PLS-R for Sudan II–IV presented excellent accuracies in predicting concentrations in standard solutions. Table 1 depicts the results of PLS-R models, including the *Q*^2^, estimated LoDs and RMSECV for all four Sudan dyes. It can be seen that excellent agreement between actual and predicted concentrations of each of four Sudan dyes were obtained from these quantitative analyses. It is also clear that the *Q*^2^ of Sudan IV was higher than other Sudan dyes.

**Figure 4:**
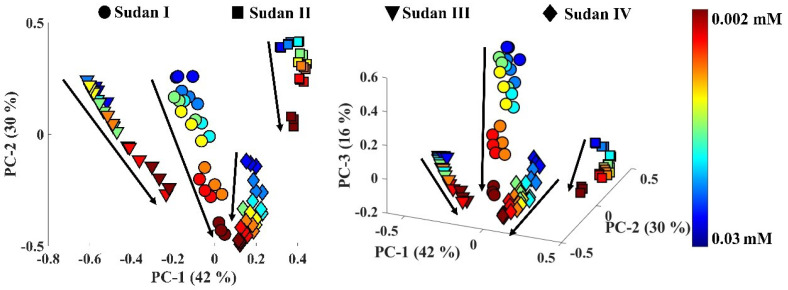
Plot of 2D (**left**) and 3D (**right**) PCA scores for all the SERS spectra of solutions of Sudan I−IV with different concentration (from 0.03 mM to 0.002 mM). A rainbow color bar represented the concentration of the four dyes, which are represented by different symbols. The values on axes in parentheses represent the total explained variance for each principal component (PC).

PCA was also applied to all SERS spectra from the four single dye dilution series and the PCA scores plot showed satisfactory discrimination between the SERS data acquired from all four Sudan dyes (Figure 4). It was also clear that trends associated with varying concentrations could be seen in the PCA scores plot as indicated by the arrows, and that although these all generally followed the same direction the exact trajectories were different.

### 3.2. Simultaneous Multiplexed Quantification of Sudan I–IV Dyes

Having established that accurate quantification was possible on all four dyes in individual mixtures the next stage was therefore to investigate the possibility of multiplexed detection and simultaneous quantification of the four dyes when they were mixed together in a quadruplex.

If we consider a quadruplex where each dye can be present at 0 to 100% of the linear range for that dye from the simplex samples at 5% interval, then each dye could be present in one of 21 concentrations. For a simplex, this is only 21 samples, but for a duplex this is 21^4^ which for an exhaustive experiment where all possible combinations are made is 194,481 possible samples! If each analysis took just 1 min then it would take nearly a year (11.25 months) to measure all samples. Clearly this is not feasible, not (chemically or environmentally) sustainable and another approach needs to be taken. To ensure there is a good coverage of concentrations of each dye in the mixtures while keeping the number of samples within a manageable range, a Latin hypercube sampling (LHS) experimental design was used to generate a set of just 90 mixtures (see Table S1 for details) which is a good representative subset of the over 194 thousand possible combinations. This resulted in a considerable decrease in sample analysis time and cost, taking just 90 min to analyse using SERS under identical conditions as those used for the simplex analyses and still be able to show the expected performance of the model applied to quadruplex mixtures.

As we were attempting to quantify four Sudan dyes simultaneously from the quadruplex mixture there are two ways to implement PLS-R. The first is to generate four separate PLS1 models-one for each of the four dyes-and this was performed using re-sampling based on 1000 bootstrap iterations. Within each bootstrap iteration, samples were split into training and test set by sampling with replacement and the model selection (i.e., to determine the optimal number of PLS components) was performed by a *k*-fold cross-validation on the training set where *k* is the number of different concentrations in the training set. The second is to generate a single PLS2 model where 4 outputs Y-variables encode the concentrations of each of the four dyes; for validation 10,000 bootstraps iterations were performed in the same way as PLS1 modelling. It was assumed that modelling four outputs simultaneously would be harder and expected higher variation in predictions with different combination of training and test set. Therefore, more iterations of re-sampling are required.

**Figure 5:**
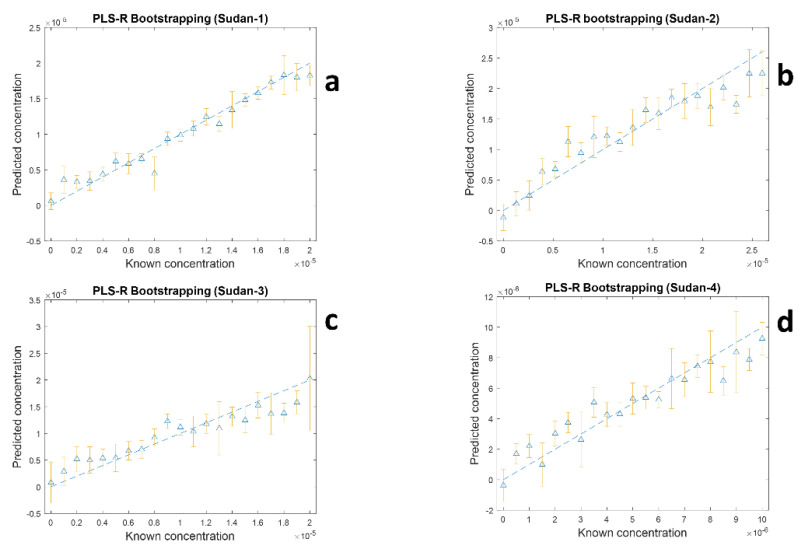
Results of PLS−R predictions vs. known concentrations of four dyes in a multiplex mixture sampled using SERS for: (**a**) Sudan I, (**b**) Sudan II, (**c**) Sudan III and (**d**) Sudan IV. In PLS−R modelling 1000 bootstrap resamples were used in each of the four PLS1 models and the plots show the average (as triangles) and the error bars the standard deviations from the 1000 test sets.

The PLS-R results using four separate PLS1 models of the 90 mixtures generated by LHS are shown in Figure 5 and the figures of merit for these four models in terms of coefficient of determination (*R*^2^ for training and *Q*^2^ for test sets) and average RMS errors from training (RMSECV) and test sets (RMSEP) from the 1000 bootstrap re-samplings are detailed in Table 2. Figure 5 and Table 2 show the results from both the training sets and test sets and this can be used to assess overall model stability as well the predictive ability of the analysis. The first thing to note for the four PLS1 models is that for each model has similar levels of goodness-of-fit for the training and test sets in terms of *R*^2^ and *Q*^2^. Similarly, the RMSECV and RMSEP for the training and test sets are similar which suggests stability of the models. Although the modelling has worked and reasonable predictions have been achieved, there is a significant variance in predictive accuracies of four dyes. The model had best predictive accuracy for Sudan I with an average *Q*^2^ of 0.8593 and RMSEP of 2.667 × 10^−^^6^ M, while Sudan III showed poorest predictive accuracy with an averaged *Q*^2^ of 0.5207 and RMSEP of 3.789 × 10^−^^6^ M. This can potentially be explained, where, according to the Lombardi and Birke [40] in SERS some selection rules, and some transitions are more substantially improved than others. Thus, depending on the binding process of the dyes to the surface of silver one dye will have preferential affinity compared to other dyes, and this will be more of an issue when coverage of the analyte on the metal surface is above a monolayer level. This has also been seen for thiols where the se analytes can associate and disassociate with the surface leading to dynamics in the sampling process [41].

Inspection of the PLS2 modelling results for the prediction of all four dyes simultaneously is also shown in Table 2. When compared to the individual models (PLS1) it can be seen that the goodness-of-fit is lower and the model errors are higher. However, these values are still good and shows that these models are also useful for predicting the levels of the four Sudan dyes in the quadruplex mixture. As reported before [42] the fact that PLS1 outperforms PLS2 in prediction accuracy is to be expected as fewer latent variables need to be optimised during the regression process and the model is therefore simpler. We note from this table that the figures of merit for PLSR predictions show that Sudan I and II are predicted with better accuracy and better linearity than Sudan III and IV (Table 2). This may be a reflection of the influence of the differences in chemical affinities of the analytes towards Au nanoparticles, and may be driven by the fact that Sudan I and II are smaller dye molecules than Sudan III and IV. However, we also note there is no direct evidence for this suggestion.

## 4. Conclusions

Sudan dyes are potential carcinogens and have been linked to numerous food safety issues. Unfortunately, these azo dyes are frequently used illegally as colorants in a wide range of foods. Although mass spectrometry coupled to chromatographic separations are used, these require the transportation of the sample to a central testing laboratory. Thus a more attractive approach is to take the instrument to the sample and effect point-of-use analysis. As a result, it is essential to have a fast and potentially portable detection tools to be able to identify various kinds of dyes and to measure their levels. In this study, gold nanoparticles were synthesized and selected as substrate for the analysis of Sudan I–IV dyes using SERS. We have shown that PCA on these SERS spectra was able to distinguish and classify the different Sudan dyes and provided evidence that trends in PCA scores space could be correlated to the Sudan I–IV concentrations. PLS-R modelling was used for quantitative analysis and showed relatively good agreement between the actual and predicted amounts of Sudan I–IV dyes both in simplex as well as quadruplex mixtures. The latter highlighting the utility of SERS for simultaneous multiplexed quantification. Finally, we estimated for the simplex mixtures that the limit of detection (LoD) for all four dyes was in the range 5.35 × 10^−5^ M to 1.84 × 10^−6^ M. This was significantly better when compared to other similar studies that reported LoD ranging from 2.5 × 10^−3^ M to 2 × 10^−4^ M [17,22,25,43].

In conclusion, the recent implementation of portable Raman systems provides quick and low-cost detection of Sudan dyes I–IV using a simple SERS substrate, and we believe this provides options for on-site testing where samples do not have to be transported back to the laboratory for analyses. With further study this methodology might also be used to study other limited or prohibited food additives and to determine ultra-low traces of certain chemical species in complex systems such as cooked food.

## Figures and Tables

**Table 1:** Results from the four PLS−R models for the individual dilution series.

	LoD (M) *	*Q* ^2^	RMSECV (M)
Sudan I	6.27 × 10^−6^	0.9286	2.5616 × 10^−6^
Sudan II	5.35 × 10^−5^	0.9206	2.0324 × 10^−6^
Sudan III	9.40 × 10^−6^	0.8676	3.4886 × 10^−6^
Sudan IV	1.84 × 10^−6^	0.9705	7.1678 × 10^−7^

* where: LoD is the limit of detection from the PLS−R models using Blanco et al. method [35]; *Q*^2^ is the coefficient of determination on test sets of the models and RMSECV is the root mean squared error from the cross validation.

**Table 2:** PLS−R results from the quadruplex mixtures.

	Sudan I	Sudan II	Sudan III	Sudan IV
Results from four individual PLS1 models *				
*R* ^2^	0.7835	0.7228	0.5645	0.6035
*Q* ^2^	0.8593	0.7255	0.5207	0.5940
RMSECV (M)	2.667 × 10^−6^	3.929 × 10^−6^	3.789 × 10^−6^	1.816 × 10^−6^
RMSEP (M)	2.150 × 10^−6^	3.908 × 10^−6^	3.953 × 10^−6^	1.816 × 10^−6^
Results from one PLS2 model with four (*Y*) outputs ^§^				
*R* ^2^	0.7303	0.6884	0.5101	0.5071
*Q* ^2^	0.8329	0.7288	0.5032	0.5459
RMSECV (M)	3.000 × 10^−6^	4.174 × 10^−6^	4.007 × 10^−6^	2.015 × 10^−6^
RMSEP (M)	2.308 × 10^−6^	3.895 × 10^−6^	4.023 × 10^−6^	1.905 × 10^−6^

Where: These data area calculated from either * 1000 or ^§^ 10,000 test sets used in the bootstrap analyses. *R*^2^ and *Q*^2^ are the average linearities calculated for the training data and test sets, respectively. RMSECV and RMSEP are the root mean squared error (RMSE) for the train/cross validation (CV) and test/prediction (P) sets.

## Data Availability

The data that support the findings of this study are available from the first author (TA) on request.

## Supplementary Materials

The following supporting information can be downloaded at: https://www.mdpi.com/article/10.3390/s22207832/s1, Figure S1: UV–Visible absorption spectra of the citrate reduced gold nanoparticles, Figure S2: (a) SERS spectra of Sudan III shows different concentrations and spectra are offset for clarity; (b) PCA scores 31 plot of Sudan III, the colours represent the concentrations, and the details are provided within the figure; (c) PC1 load-32 ings plot (green), with Sudan III (blue) and matrix, aggregation agent (0.5 M NaCl) and gold nanoparticles, (in brown 33 and multiplied by −1 (i.e., inverted) for clarity); (d) PLS-R predictions of Sudan III, these models used double-cross vali-34 dation., Figure S3: (a) SERS spectra of Sudan III shows different concentrations and spectra are offset for clarity; (b) PCA scores 31 plot of Sudan III, the colours represent the concentrations, and the details are provided within the figure; (c) PC1 load-32 ings plot (green), with Sudan III (blue) and matrix, aggregation agent (0.5 M NaCl) and gold nanoparticles, (in brown 33 and multiplied by −1 (i.e., inverted) for clarity); (d) PLS-R predictions of Sudan III, these models used double-cross vali-34 dation, Figure S4: (a) SERS spectra of Sudan IV shows different concentrations and spectra are offset for clarity; (b) PCA scores 39 plot of Sudan IV, the colours represent the concentrations, and the details are provided within the figure; (c) PC1 load-40 ings plot (green), with Sudan IV (blue) and matrix, aggregation agent (0.5 M NaCl) and gold nanoparticles, (in brown 41 and multiplied by −1 (i.e., inverted) for clarity); (d) PLS-R predictions of Sudan IV, these models used double-cross vali-42 dation, Figure S5: SEM micrographs acquired from gold nanoparticles bound to stainless steel substrate. The scale bars and 55 magnification are inset in each of the images. Figure S6: SERS spectra acquired from quadruplex mixtures. Mixture 1 (sample 1 in Table S1); Mixture 2 (sample 2 in 58 Table S1); Mixture 3 (sample 3 in Table S1); Mixture 4 (sample 4 in Table S1); Mixture 5 (sample 5 in Table S1); Table S1: Latin Hypercubic Sampling of combination Sudan dyes I–IV.
