# Supplementary material for: Simultaneous Multiplexed Quantification of Banned Sudan Dyes Using Surface Enhanced Raman Scattering and Chemometrics
Source: Sensors (Basel). 2022 Oct 15;22(20):7832. doi: 10.3390/s22207832 (PMC9611880; doi:10.3390/s22207832)
Supplement: Supplementary file 1 [file sensors-22-07832-s001.zip › sensors-1853831-supplementary.pdf]

sensors

MDPI

*Article*

# *Simultaneous multiplexed quantification of banned Sudan dyes using surface enhanced Raman scattering and chemometrics*

**Taghrid S. Alomar[1,2], Najla AlMasoud[1,2], Yun Xu[2], Cassio Lima[2], Baris Akbali[3,4], Simon Maher[3], Royston Goodacre[2]**

[1] Department of Chemistry, College of Science, Princess Nourah bint Abdulrahman University, Riyadh 11671, Saudi Arabia
[2] Centre for Metabolomics Research, Department of Biochemistry and Systems Biology, Institute of Systems, Molecular and Integrative Biology, University of Liverpool, Biosciences Building, Crown Street, Liverpool L69 7ZB, UK
[3] Department of Electrical Engineering and Electronics, University of Liverpool, Brownlow Hill, Liverpool, L69 3GJ, UK
[4] Department of Engineering and System Science, National Tsing Hua University, Hsinchu 30013, Taiwan

* Correspondence: roy.goodacre@liverpool.ac.uk;

## Supplementary Materials

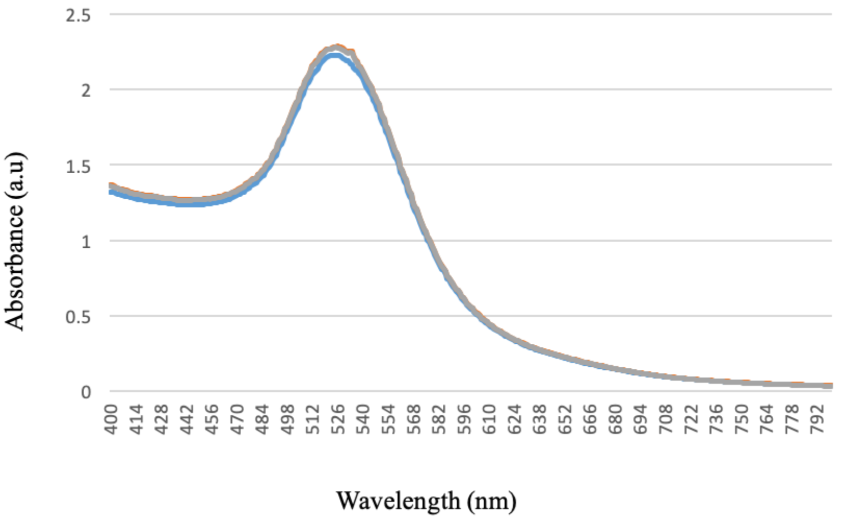


**Figure S1**: UV–Visible absorption spectra of the citrate reduced gold nanoparticles.

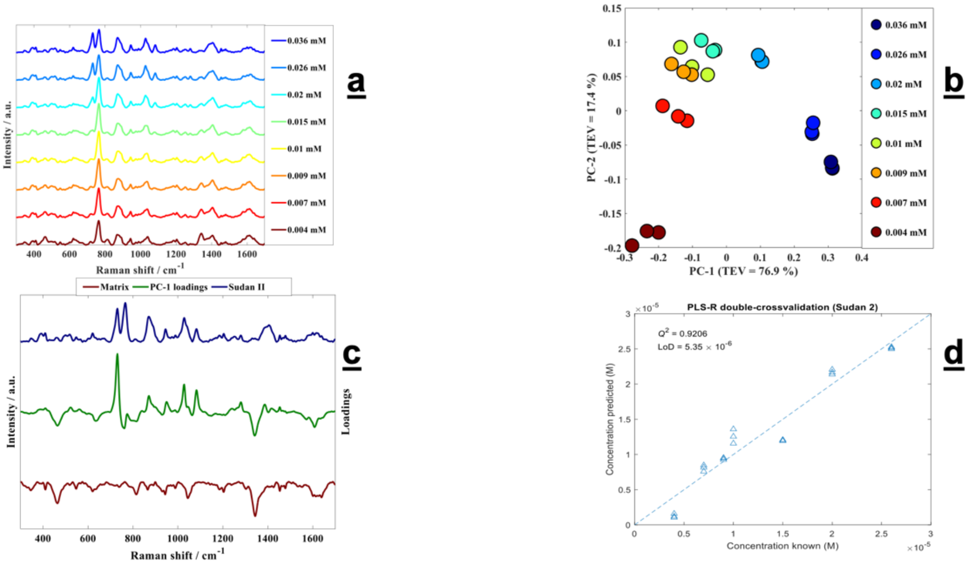


**Figure S2:** (a) SERS spectra of Sudan II shows different concentrations and spectra are offset for clarity; (b) PCA scores plot of Sudan II, the colours represent the concentrations, and the details are provided within the figure; (c) PC1 loadings plot (green), with Sudan II (blue) and matrix, aggregation agent (0.5M NaCl) and gold nanoparticles, (in brown and multiplied by -1 (i.e. inverted) for clarity); (d) PLS-R predictions of Sudan II, these models used double-cross validation**.**

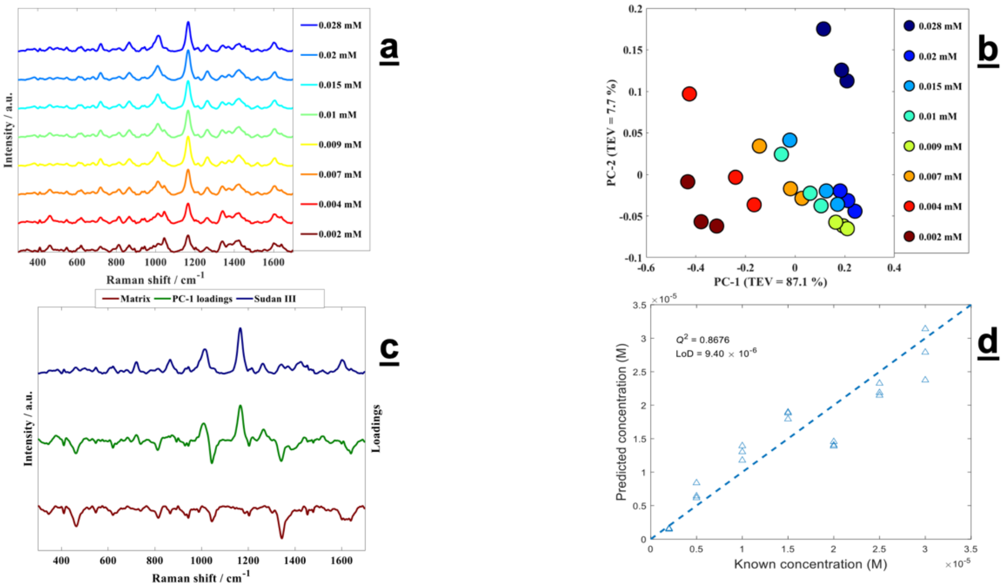


**Figure S3:** (a) SERS spectra of Sudan III shows different concentrations and spectra are offset for clarity; (b) PCA scores plot of Sudan III, the colours represent the concentrations, and the details are provided within the figure; (c) PC1 loadings plot (green), with Sudan III (blue) and matrix, aggregation agent (0.5M NaCl) and gold nanoparticles, (in brown and multiplied by -1 (i.e. inverted) for clarity); (d) PLS-R predictions of Sudan III, these models used double-cross validation.

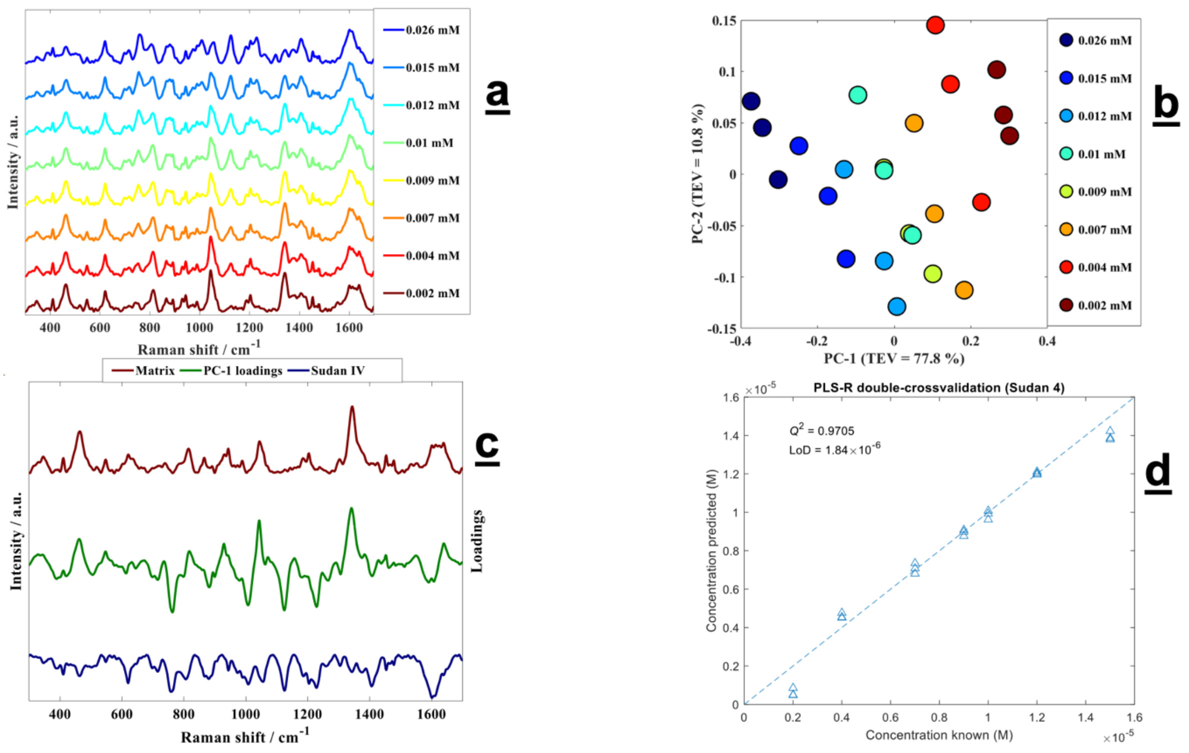


**Figure S4**: (a) SERS spectra of Sudan IV shows different concentrations and spectra are offset for clarity; (b) PCA scores plot of Sudan IV, the colours represent the concentrations, and the details are provided within the figure; (c) PC1 loadings plot (green), with Sudan IV (blue) and matrix, aggregation agent (0.5M NaCl) and gold nanoparticles, (in brown and multiplied by -1 (i.e. inverted) for clarity); (d) PLS-R predictions of Sudan IV, these models used double-cross validation**.**

**Table S1**: Latin Hypercubic Sampling of combination Sudan dyes I-IV

| Sample | Sudan I | Sudan II | Sudan III | Sudan IV | Sample | Sudan I | Sudan II | Sudan III | Sudan IV |
|---|---|---|---|---|---|---|---|---|---|
| 1 | 0 | 60 | 30 | 50 | 46 | 50 | 60 | 60 | 65 |
| 2 | 0 | 65 | 50 | 75 | 47 | 50 | 80 | 10 | 95 |
| 3 | 0 | 85 | 90 | 15 | 48 | 55 | 5 | 80 | 20 |
| 4 | 5 | 5 | 85 | 20 | 49 | 55 | 70 | 60 | 100 |
| 5 | 5 | 15 | 5 | 80 | 50 | 55 | 75 | 5 | 30 |
| 6 | 5 | 15 | 45 | 10 | 51 | 55 | 85 | 30 | 80 |
| 7 | 5 | 100 | 10 | 70 | 52 | 55 | 90 | 40 | 55 |
| 8 | 10 | 15 | 60 | 25 | 53 | 60 | 0 | 95 | 50 |
| 9 | 10 | 20 | 75 | 15 | 54 | 60 | 50 | 15 | 25 |
| 10 | 10 | 25 | 45 | 5 | 55 | 60 | 65 | 40 | 5 |
| 11 | 10 | 85 | 30 | 70 | 56 | 60 | 70 | 15 | 45 |
| 12 | 15 | 20 | 70 | 60 | 57 | 60 | 90 | 90 | 85 |
| 13 | 15 | 45 | 65 | 75 | 58 | 65 | 10 | 65 | 10 |
| 14 | 15 | 50 | 40 | 35 | 59 | 65 | 75 | 35 | 5 |
| 15 | 15 | 55 | 95 | 60 | 60 | 65 | 95 | 35 | 35 |

| | | | | | | | | | |
|---|---|---|---|---|---|---|---|---|---|
| 16 | 15 | 80 | 100 | 45 | 61 | 70 | 5 | 0 | 45 |
| 17 | 20 | 10 | 25 | 90 | 62 | 70 | 40 | 30 | 20 |
| 18 | 20 | 20 | 45 | 55 | 63 | 70 | 60 | 75 | 10 |
| 19 | 20 | 20 | 80 | 95 | 64 | 70 | 80 | 100 | 65 |
| 20 | 20 | 65 | 50 | 55 | 65 | 70 | 90 | 85 | 30 |
| 21 | 25 | 25 | 45 | 30 | 66 | 75 | 0 | 80 | 80 |
| 22 | 25 | 55 | 60 | 45 | 67 | 75 | 35 | 90 | 80 |
| 23 | 25 | 80 | 55 | 50 | 68 | 75 | 40 | 10 | 95 |
| 24 | 25 | 85 | 25 | 15 | 69 | 75 | 95 | 15 | 5 |
| 25 | 30 | 20 | 85 | 20 | 70 | 75 | 100 | 55 | 45 |
| 26 | 30 | 30 | 5 | 90 | 71 | 80 | 30 | 85 | 60 |
| 27 | 30 | 30 | 15 | 100 | 72 | 80 | 70 | 80 | 40 |
| 28 | 30 | 35 | 20 | 75 | 73 | 80 | 75 | 55 | 25 |
| 29 | 30 | 45 | 65 | 65 | 74 | 80 | 75 | 90 | 65 |
| 30 | 30 | 45 | 75 | 70 | 75 | 80 | 80 | 20 | 85 |
| 31 | 35 | 65 | 5 | 60 | 76 | 85 | 30 | 75 | 85 |
| 32 | 35 | 85 | 40 | 40 | 77 | 85 | 55 | 10 | 25 |
| 33 | 35 | 90 | 30 | 85 | 78 | 85 | 60 | 50 | 75 |
| 34 | 40 | 5 | 95 | 5 | 79 | 85 | 70 | 0 | 70 |
| 35 | 40 | 10 | 65 | 30 | 80 | 90 | 10 | 5 | 0 |
| 36 | 40 | 25 | 70 | 15 | 81 | 90 | 35 | 70 | 0 |
| 37 | 40 | 50 | 35 | 95 | 82 | 90 | 40 | 60 | 40 |
| 38 | 40 | 95 | 85 | 85 | 83 | 90 | 45 | 95 | 40 |
| 39 | 45 | 30 | 20 | 95 | 84 | 95 | 45 | 30 | 35 |
| 40 | 45 | 35 | 40 | 65 | 85 | 95 | 50 | 55 | 15 |
| 41 | 45 | 50 | 70 | 90 | 86 | 95 | 70 | 75 | 55 |
| 42 | 45 | 55 | 25 | 35 | 87 | 95 | 95 | 15 | 35 |
| 43 | 50 | 5 | 25 | 50 | 88 | 95 | 95 | 20 | 90 |
| 44 | 50 | 20 | 55 | 20 | 89 | 100 | 15 | 50 | 55 |
| 45 | 50 | 40 | 35 | 75 | 90 | 100 | 35 | 95 | 10 |

The values in the table are percentages that represent those of the different ranges from each of the dyes:

Sudan I is 0.048 – 0.002 mM

Sudan II: 0.036 – 0.004 mM

Sudan III: 0.028 – 0.002 mM

Sudan IV: 0.026 – 0.002 mM

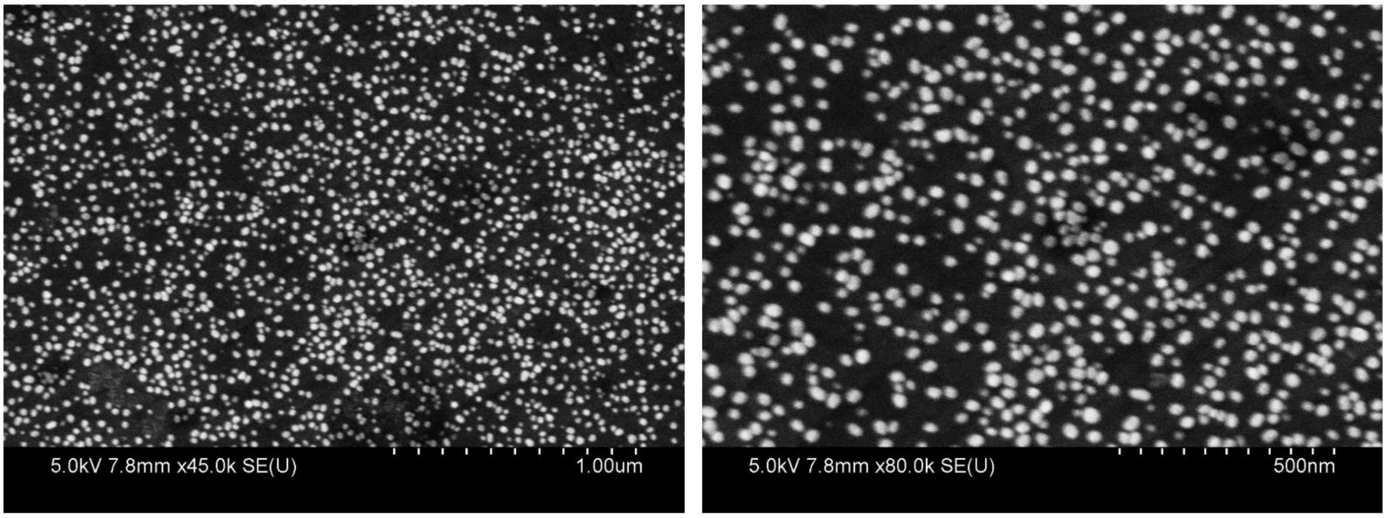


**Figure S5:** SEM micrographs acquired from gold nanoparticles bound to stainless steel substrate. The scale bars and magnification are inset in each of the images.

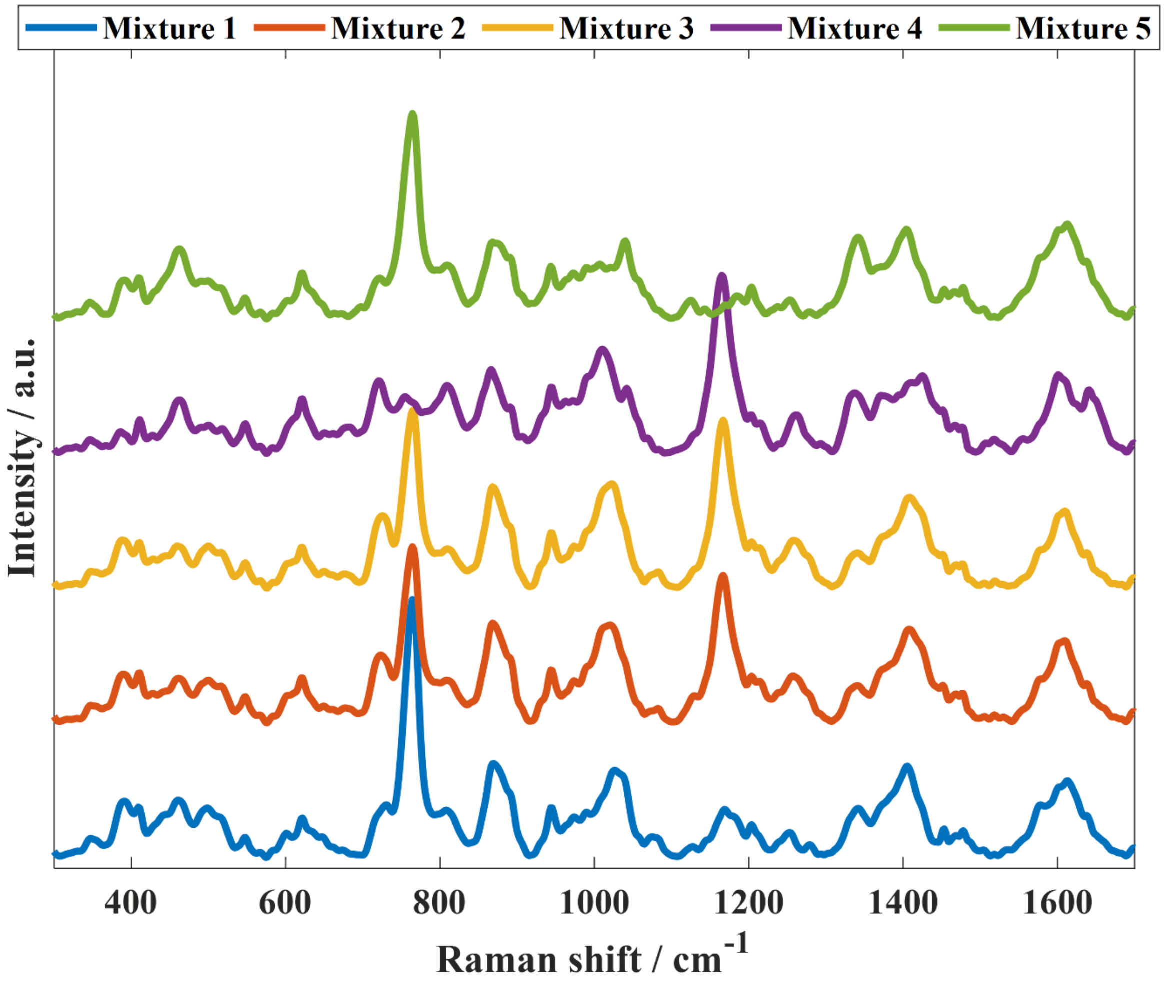


**Figure S6:** SERS spectra acquired from quadruplex mixtures. Mixture 1 (sample 1 in table S1); Mixture 2 (sample 2 in table S1); Mixture 3 (sample 3 in table S1); Mixture 4 (sample 4 in table S1); Mixture 5 (sample 5 in table S1).
